# Reduced Expression of Fumarate Hydratase in Clear Cell Renal Cancer Mediates HIF-2α Accumulation and Promotes Migration and Invasion

**DOI:** 10.1371/journal.pone.0021037

**Published:** 2011-06-14

**Authors:** Sunil Sudarshan, Karthigayan Shanmugasundaram, Susan L. Naylor, Shu Lin, Carolina B. Livi, Christine F. O'Neill, Dipen J. Parekh, I-Tien Yeh, Lu-Zhe Sun, Karen Block

**Affiliations:** 1 Department of Urology, University of Texas Health Sciences Center at San Antonio, San Antonio, Texas, United States of America; 2 Department of Cellular and Structural Biology, University of Texas Health Sciences Center at San Antonio, San Antonio, Texas, United States of America; 3 Department of Molecular Medicine, University of Texas Health Sciences Center at San Antonio, San Antonio, Texas, United States of America; 4 Department of Epidemiology and Biostatistics, University of Texas Health Sciences Center at San Antonio, San Antonio, Texas, United States of America; 5 Department of Pathology, University of Texas Health Sciences Center at San Antonio, San Antonio, Texas, United States of America; 6 Department of Medicine, University of Texas Health Sciences Center at San Antonio, San Antonio, Texas, United States of America; 7 Audie L. Murphy Memorial Hospital Division, South Texas Veterans Health Care System, San Antonio, Texas, United States of America; University of Illinois at Chicago, United States of America

## Abstract

Germline mutations of *FH*, the gene that encodes for the tricarboxylic acid TCA (TCA) cycle enzyme fumarate hydratase, are associated with an inherited form of cancer referred to as Hereditary Leiomyomatosis and Renal Cell Cancer (HLRCC). Individuals with HLRCC are predisposed to the development of highly malignant and lethal renal cell carcinoma (RCC). The mechanisms of tumorigenesis proposed have largely focused on the biochemical consequences of loss of FH enzymatic activity. While loss of the tumor suppressor gene *von Hippel Lindau* (*VHL*) is thought to be an initiating event for the majority of RCCs, a role for *FH* in sporadic renal cancer has not been explored. Here we report that FH mRNA and protein expression are reduced in clear cell renal cancer, the most common histologic variant of kidney cancer. Moreover, we demonstrate that reduced *FH* leads to the accumulation of hypoxia inducible factor- 2α (HIF-2α), a transcription factor known to promote renal carcinogenesis. Finally, we demonstrate that overexpression of FH in renal cancer cells inhibits cellular migration and invasion. These data provide novel insights into the tumor suppressor functions of FH in sporadic kidney cancer.

## Introduction

In 2010, over 57,000 men and women will be diagnosed with renal cell carcinoma (RCC) and 13,000 individuals will die of this disease [1]. Although survival for patients with localized disease is high, patients with advanced disease face a poor prognosis despite recently introduced targeted agents. Though loss of the *von Hippel Lindau* (*VHL*) tumor suppressor gene is thought to be an initiating event for the majority of RCCs [2], little is known about subsequent genetic events and their respective impact on tumorigenesis. Elucidation of these pathways will identify novel therapeutic targets as well as facilitate biomarker development that may have both diagnostic and prognostic significance.

Germline mutations of *FH* are associated with an inherited form of renal cancer referred to as Hereditary Leiomyomatosis and Renal Cell Cancer (HLRCC) [3], [4]. *FH* encodes the tricarboxylic acid cycle enzyme fumarate hydratase (also referred to as fumarase) which catalyzes the hydration of fumarate to form malate. Individuals with HLRCC are predisposed to the development of leiomyomas of the skin and uterus in addition to highly malignant and lethal RCC. The mechanisms of tumorigenesis proposed have largely focused on the biochemical consequences of loss of FH enzymatic activity. It has been proposed that loss of FH leads to fumarate accumulation and promotes a pseudohypoxic state in which hypoxia response pathways are aberrantly activated despite normoxic conditions [5]. Fumarate has been shown to inhibit proline hydroxylation of the hypoxia inducible factors HIF-1α and HIF-2α which are catalyzed by a family of enzymes referred to as the HIF prolyl hydroxylases (PHDs) [5]. In their unhydroxylated form, HIFαs avoid recognition by the E3 ubiquitin ligase VHL (which targets these proteins for proteosomal degradation) and are thus stabilized [6], [7], [8], [9]. Under these conditions either HIF-1α or HIF-2α are able to heterodimerize with the constitutively expressed protein HIF-1β, also referred to as ARNT (recently reviewed [10]). This heterocomplex is able to transcriptionally activate several genes including *VEGF* and other growth factors that may be pro-tumorigenic when dysregulated. Pseudohypoxia has also been implicated in the most common variant of RCC, clear cell carcinoma (ccRCC), in which loss of *VHL* is a common genetic event [11]. As expected, elevated levels of HIF-1α and/or HIF-2α are noted in clear cell renal cancers [12], [13]. Interestingly, several lines of evidence indicate that HIF-2α as opposed to HIF-1α, is critical to RCC formation and/or progression [14], [15], [16].

While *VHL* loss is clearly critical to HIF-2α stabilization, alternate mechanisms, besides the prevention of degradation, may play a role in the maintenance of HIF-2α in renal cancer. Previous work by Block *et al.* established a role for reactive oxygen species (ROS) generated by NADPH oxidases in maintaining HIF-2α protein expression through an AKT-dependent mRNA translational mechanism in VHL-deficient cells [17]. In addition, mTOR signaling complex 2 (mTORC2), a known activator of AKT signaling, has been shown to promote HIF-2α accumulation in *VHL* null renal carcinoma cells [18]. More recently, treatment of RCC cells with a dual PI3K/mTOR inhibitor suppressed the expression of HIF-2α [19]. These data support the notion that ongoing HIF-2α synthesis is critical to the maintenance of this oncogenic transcription factor in renal cancer cells.

*FH* mutations have primarily been linked to papillary type II renal cancer, a histologic variant that accounts for less than 10% of all renal cancers [20]. *FH* mutations have not been identified in sporadic clear cell renal cancer. However, a recent report has linked *FH* to the development of clear cell renal cancer in a patient with a germline mutation of *FH* [21]. To date, the expression and function of *FH* in ccRCC has not examined. Therefore, we investigated the role of FH in sporadic clear cell renal cancer.

## Results

### Reduced expression of FH in clear cell renal carcinoma

**Figure 1:**
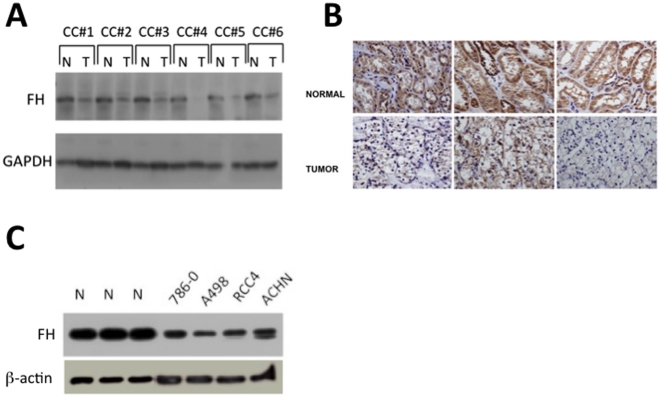
FH protein expression id reduced in clear cell renal cancer. A) Protein was isolated from clear cell (CC) tumor samples (T) in addition to matched normal renal parenchyma (N). Proteins were immunoblotted for FH protein levels. GAPDH immunoblot is included as a loading control. B) Immunohistochemical staining for FH was performed on patient-matched tumor/normal pairs. Images were obtained with a 40× objective lens. C) FH protein levels in RCC lines relative to normal kidney. Actin is included as a loading control.

**Figure 2:**
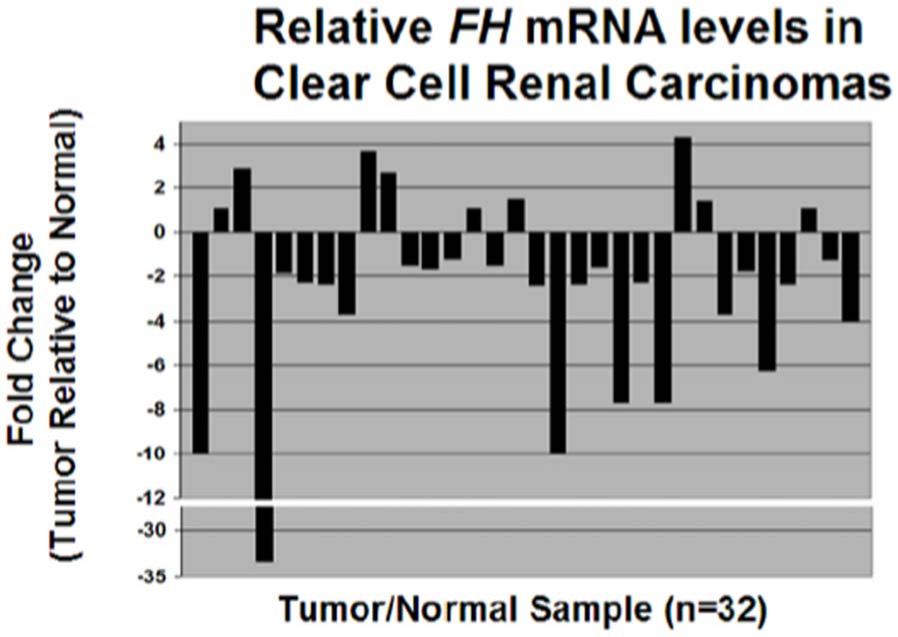
FH mRNA expression is reduced in clear cell kidney cancer. mRNA levels of *FH* were determined in a separate set of tumor samples relative to patient matched normal renal parenchyma with real time RT-PCR (p = 0.004). Expression levels were normalized to 18 s rRNA levels prior to comparative analysis.

FH expression in ccRCC has yet to be explored. Therefore, we first examined the protein expression of FH in a panel of human clear cell renal tumors and patient-matched normal renal parenchyma. Immunoblot analysis of tissue lysates demonstrated a marked reduction of FH protein levels in the tumors as compared to normal adjacent tissue (Figure 1A). To confirm our findings, we performed immunohistochemical staining for FH on patient matched tumor/normal pairs. These results correlated with our immunoblotting results in that staining for FH was less in tumors relative to control renal tissue (Figure 1B). We next examined FH protein levels in a panel of established ccRCC cell lines (786-O, A498, RCC4, and ACHN). All cultured cell lines demonstrated reduced FH protein levels relative to normal kidney (Figure 1C). We then examined mRNA expression levels of *FH* in tumor tissue as compared to patient-matched normal renal tissue in specimens from the Cooperative Human Tissue Network (NCI). Quantitative real time RT-PCR demonstrated reduced *FH* mRNA levels in tumor tissue as compared with normal tissue (Figure 2). Analysis of mRNA levels reveals that over 70% of the tumor samples demonstrated reduced *FH* mRNA levels relative to normal matched renal parenchyma. Moreover, 15/32 patient samples (47%) demonstrated a greater than twofold reduction in *FH* mRNA levels in tumor samples relative to normal control. Overall, the average reduction in *FH* mRNA levels was 2.9 fold. This difference was determined to be statistically significant. These results demonstrate reduced expression of *FH* at the mRNA and protein levels in ccRCC.

### FH modulates HIF-2α levels

**Figure 3:**
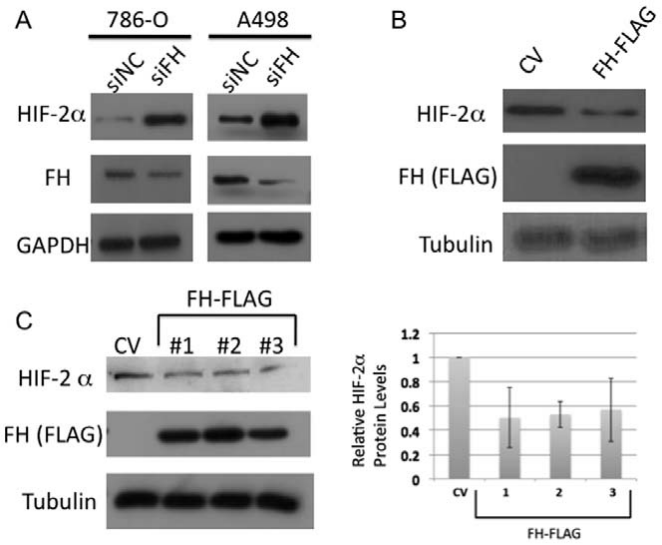
FH expression impacts HIF-2α levels. A) *VHL* null 786-O and A498 cells were transected with siRNA to FH and scramble control (siNC). Forty-eight hours following transfection, protein lysates were analyzed by immunoblotting for the indicated proteins. B) 786-O cells were transiently transfected with control vector (CV) and vector containing FLAG tagged FH. Forty-eight hours following transfection, protein lysates were analyzed by immunoblotting for the indicated proteins. FLAG immunoblot indicates successful expression of the transgene. C) (Left) 786-O cells were stably transfected with CV and FH-FLAG. After selection in puromycin, single cell clones were harvested and screened for FLAG expression. HIF-2α levels were measured in FH-FLAG expressing clones relative to CV transfected cells. Densitometry of the bands is quantitatively displayed on the right. Mean relative values +/− standard deviation were obtained with ImageJ software from independent experiments.

High levels of fumarate in FH-deficient RCC play a role in stabilizing HIF-2α protein expression through inhibition of proline hydroxylase activity thereby preventing VHL recognition. Based on these data, we hypothesized that loss of FH should have no impact on HIF protein levels in *VHL* null cell lines. However, we found that siRNA-mediated knockdown of FH resulted in a further increase of HIF-2α protein levels in two ccRCC lines which are *VHL* null (786-O and A498) (Figure 3A). In support of these findings, transient overexpression of FLAG-tagged FH (FH-FLAG) reduced HIF-2α levels in 786-O cells (Figure 3B). Together, this suggests that FH modulates HIF-2α protein expression in the absence of VHL.

To further elucidate the mechanism by which FH regulate HIF-2α protein expression, we created stable cells lines overexpressing FH-FLAG in VHL-deficient 786-O cells. We identified 3 clones that stably expressed the FH-FLAG construct. All 3 clones demonstrated reduced HIF-2α levels as compared with control vector transfected cells (Figure 3C). We initially considered whether the effects on HIF-2α were transcriptionally mediated, however we did not detect reductions in HIF-2α mRNA levels with FH overexpression (data not shown).

### FH loss activates AKT signaling

**Figure 4:**
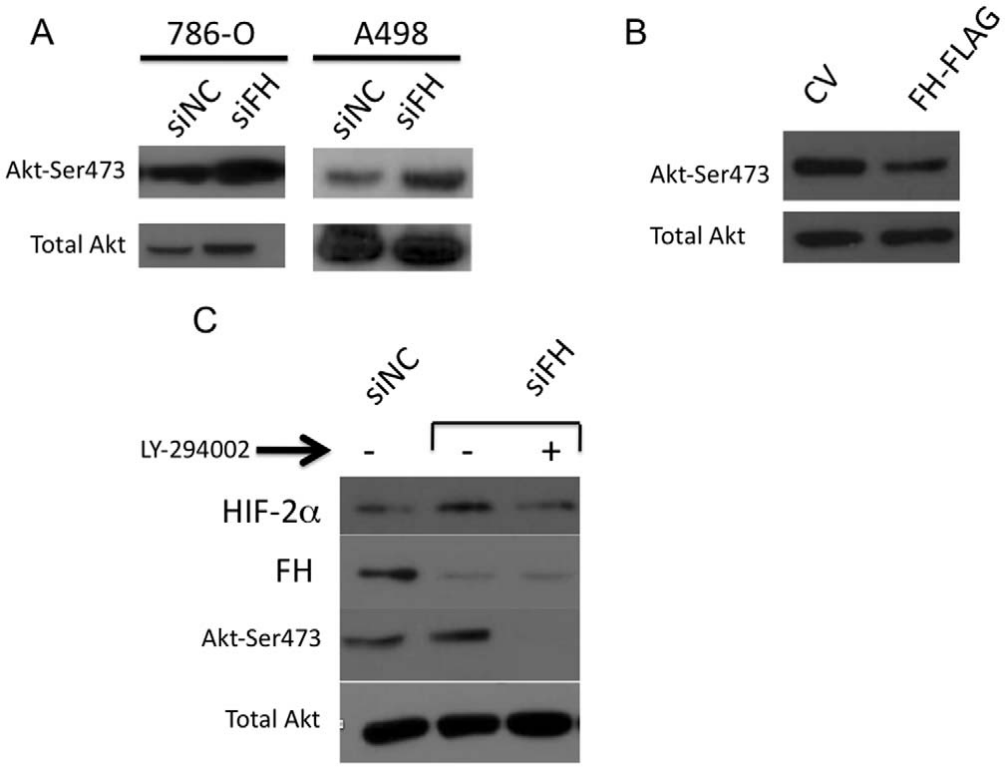
FH expression impacts AKT signaling. A) *VHL* null 786-O and A498 cells were transfected with siRNA to FH and scramble control (siNC). Forty-eight hours following transfection, protein lysates were analyzed by immunoblotting for total AKT and ser473 phospho-AKT. B) 786-O cells were transiently transfected with control vector (CV) and vector containing FLAG tagged FH. Forty-eight hours following transfection, protein lysates were analyzed by immunoblotting for the indicated proteins. C) 786-O cells were transfected with the indicated siRNA. Twenty-four hours following transfection, media of the cells was replaced with media containing PI3K inhibitor LY-294002 (6.25 µM). Cells were then harvested 24 hours later and protein lysates were subjected to immunoblot analysis for the indicated proteins.

Recent reports have implicated PI3K/AKT signaling in maintaining HIF-2α protein expression in *VHL* null cells through a translational mechanism [17], [18], [19], [22]_ENREF_17. Based on these reports, we examined AKT signaling with FH modulation. We found that siRNA-mediated knockdown of *FH* in both 786-O and A498 cells results in increased AKT phosphorylation on serine 473 of AKT (Figure 4A). Correspondingly, overexpression of FH lowered phospho-AKT levels in 786-O cells (Figure 4B) indicating that FH levels inversely correlate with AKT signaling. We next examined the effects of PI3K inhibition on FH-dependent HIF-2α protein expression. Consistent with our previous findings, FH knockdown activated AKT signaling and increased HIF-2α levels compared to scramble transfected cells (siNC) (Figure 4C). However, cotreatment of transfected cells with the PI3K inhibitor LY-294002 blocked the increase in HIF-2α associated with FH knockdown (Figure 4C). Together, this suggests that FH maintains HIF-2α protein expression through mechanisms dependent on PI3K and AKT signaling.

### FH expression mediates cell migration and invasion

**Figure 5:**
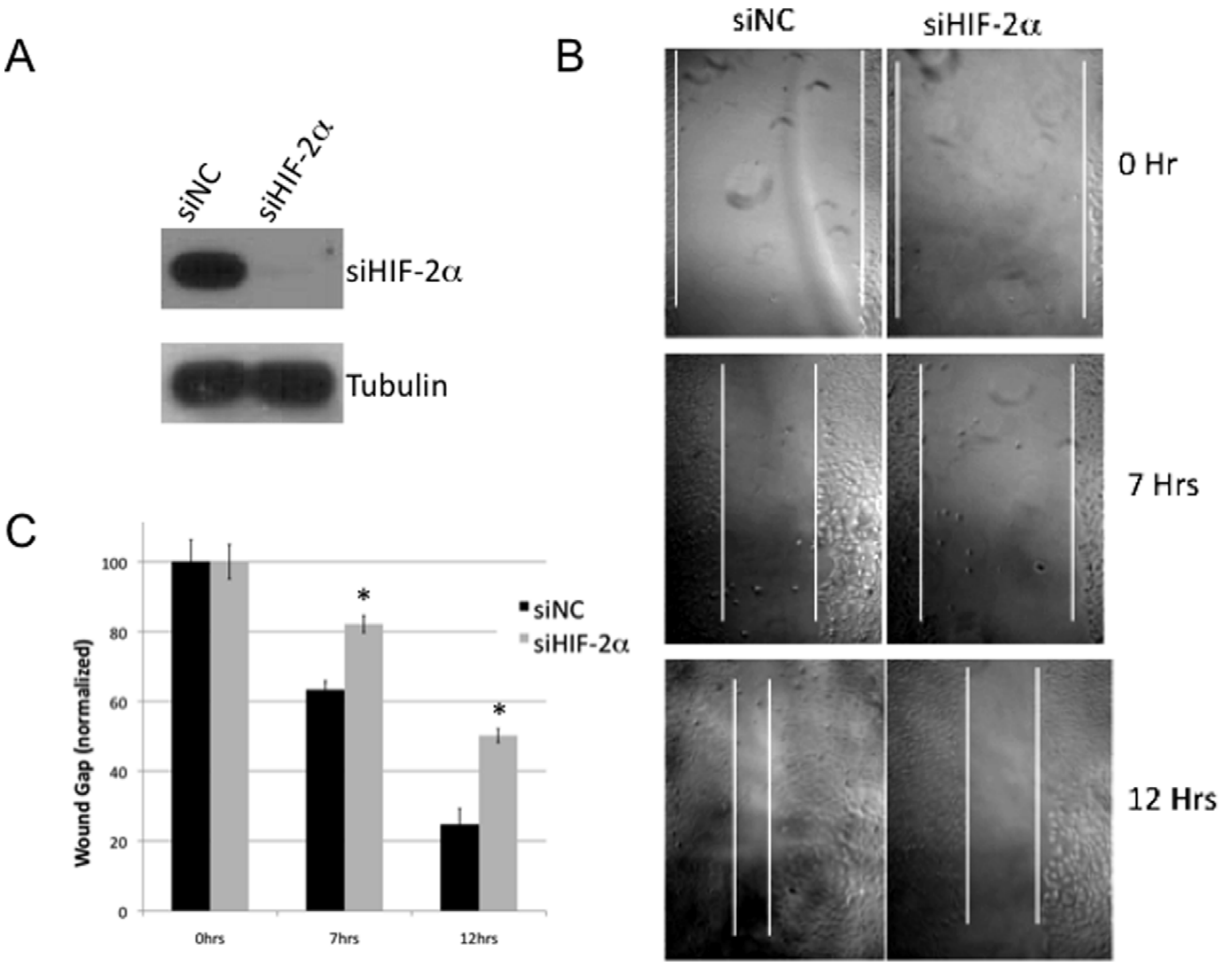
HIF-2α promotes migration in RCC cells. 786-O RCC cells were transfected with scramble control siRNA (siNC) or siRNA to HIF-2α. Western blotting results of whole cell extracts are demonstrated in panel A. B) Wound healing assay was performed in transfected cells. Images were serially taken at the indicated time point following “wound” induction. Results are graphically displayed in panel C. Asterisks (*) indicate statistical significance with p<0.05 relative to scramble control transfected cells.

**Figure 6:**
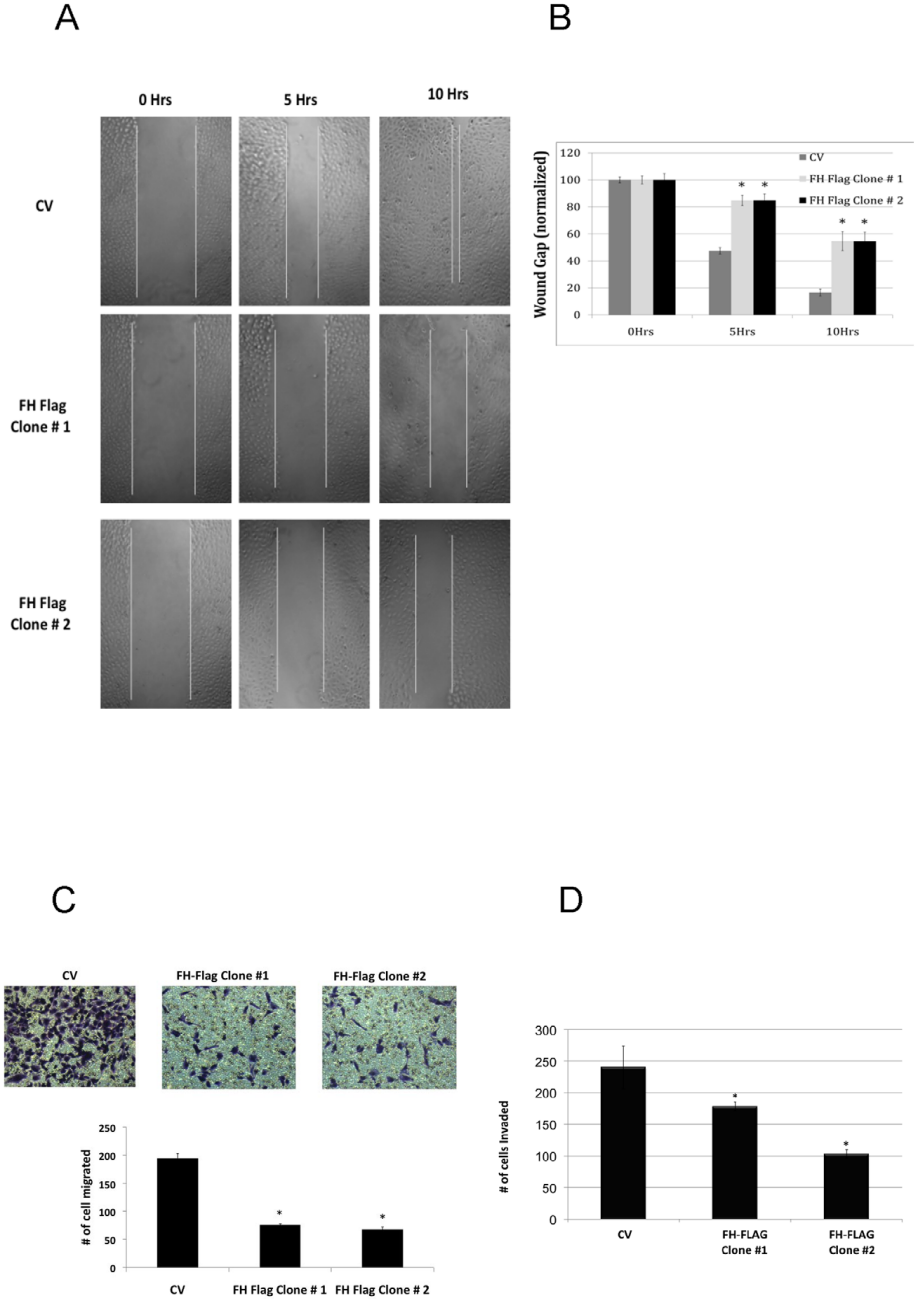
Migration and Invasion assays in 786-O cells transfected with control vector (CV) or FH-FLAG. A) Wound healing assay images at the indicated time points. B) Wound width distance at the indicated time points. C) Chamber cell migration assay of the indicated clones. Cells were seeded in serum free media with 10% FBS used as the chemotractant. Migrated cell counts of clones at 48 hours from initial seeding. Asterisks (*) indicate statistical significance with p<0.05 relative to control vector transfected cells. D) Matrigel invasion assay with 10% FBS media as the chemotractant. Asterisks (*) indicate statistical significance with p<0.05 relative to control vector transfected cells.

The biological consequences of FH overexpression in RCC cells were next examined. *FH* null tumors are highly invasive and often metastatic tumors [20], and HIF-2α has previously been implicated in this cellular process [23]. Therefore, we investigated the role of FH in cellular migration and invasion in ccRCC. We find that knockdown of HIF-2α with siRNA in 786-O RCC cells diminished cellular motility as determined by wound healing assay compared to scramble transfected cells (Figures 5A and 5B). Quantification of these results are provided in Figure 5C. While almost 80% of the wound gap was closed in control transfected cells by 12 hours, 50% of the wound gap remained in HIF-2α knockdown cells. Given these data, we examined wound healing in FH overexpressing 786-O subclones as compared with control vector transfected cells. Both FH overexpressing clones had significantly reduced wound closure as compared with control vector transfected cells, suggesting that loss of FH contributes to migration in RCC (Figure 6A). In control vector transfected cells, the wound width gap was almost completely closed by 10 hours. In contrast, FH overexpressing cells closed the wound gap by only half by 10 hours indicating reduced cellular migration was a result of FH overexpression. These results are displayed graphically (Figure 6B). To further corroborate these data, we examined cellular migration utilizing a chamber assay with 10% fetal bovine serum as the chemotractant. 786-O vector control cells were significantly more migratory than FH overexpressing clones (Figure 6C). Finally, overexpression of FH in RCC cells reduced their invasive ability as determined by matrigel invasion assay (Figure 6D). Taken together, these data demonstrate that loss of FH expression enhances the migratory and invasive ability of RCC cells.

## Discussion

In this report, we demonstrate for the first time reduced FH expression at the mRNA and protein levels in clear cell renal carcinoma, the most common histological variant that accounts for the majority of kidney cancers. These findings are significant as previous studies did not identify *FH* mutations in RCC lines as well as primary RCC specimens [24]. The mechanisms by which mRNA levels of *FH* are reduced are under current investigation. Hypermethylation may be one mechanism by which *FH* expression is suppressed. Dulaimi *et al.* did not identify hypermethylation in a CpG island of the *FH* promoter in a panel of papillary RCCs [25]. However, no studies to date have examined *FH* methylation status in ccRCC. While hypermethylation is a means of tumor suppressor gene silencing, alternate mechanisms may also account for the reduced expression of *FH* we have identified. Recent interest has focused on the role of microRNA (miRNA) in gene regulation where reports suggests that genes involved in oxidative phosphorylation may be subject to regulation by miRNAs [26]. Clearly, these possibilities warrant further investigation given the biological significance of our findings.

We have previously demonstrated that reintroduction of wild type *FH* into a *FH* deficient tumor cell line results in a marked reduction in nuclear HIF-1α levels [27]. Moreover, siRNA mediated knockdown of FH has been shown to increase HIF-1α levels in A549 lung carcinoma cells which express VHL [5]. The findings from these studies, as well as other biochemical studies, suggest that the principle mechanism by which this occurs is through HIF protein stabilization via inhibition of prolyl hydroxylase activity as a result of FH loss. These findings would therefore presume that the effect of FH on HIF would only be evident in the presence of VHL. However, our findings are quite novel in that they indicate that FH can modulate HIF-2α independently of VHL. VHL-independent pathways that mediate HIF degradation have been reported. In particular, Hsp90 and RACK1 have previously been shown to modulate HIF-1α degradation [28]. Therefore, it is possible that a similar mechanism mediates HIF-2α degradation as well. Despite stabilization via inhibition of protein degradation, there is growing evidence that alternate pathways play a role in maintaining HIF-2α protein levels in the absence of VHL. Block *et al.* demonstrated that elevated cellular reactive oxygen species, mediated by p22-phox based Nox oxidases, maintain HIF-2α protein levels in RCC cells through an AKT/4E-BP1 mRNA translational dependent mechanism [17], [22]. Correspondingly, phosphorylation of AKT and 4E-BP1 are enhanced in human RCC tissue relative to normal parenchymal tissue [22]. More recently, Toschi *et al.* found that AKT activation, via signaling through the mTOR signaling complex 2 (mTORC2), was required to maintain HIF-2α in VHL null cells [18]. AKT activation is a common signaling node in cancer and alternate mechanisms may lead to AKT activation in RCC including loss of FH.

The mechanism by which AKT signaling is activated by FH loss is under current investigation. We have previously demonstrated that loss of FH in renal epithelial cells results in elevated cellular oxidative stress [27]. Hence, ROS may be a contributor to the elevated HIF-2α levels upon FH knockdown. Alternatively, the effects of FH loss may be unrelated to its role in the TCA cycle. It is well established that FH also exists in an extramitochondrial, cytosolic form. At this time, very little is known about the function of this form of FH. However, recent evidence provided by O'Flaherty *et al.* indicates that loss of extramitochondrial FH may contribute to HIF stabilization [29]. In addition, cytosolic FH has been implicated in the DNA damage response [30]. Upon DNA damage, cytosolic FH has been shown to translocate into the nucleus. The mechanism by which FH participates in the DNA damage response remains unclear. However, there is certainly the possibility that FH and the metabolites it interacts with may regulate the function of other proteins, potentially within the nucleus. Interestingly, AKT has also been shown to function in the nucleus [31]. Given our data, as well as these recent reports, targeting AKT mediated signaling pathways, either at the level of AKT or upstream, may prove to be of therapeutic benefit for renal cancer. This is in concordance with recent data demonstrating the *in vitro* and *in vivo* efficacy of a dual PI3K/mTOR inhibitor in RCC [19].

Interestingly, there is precedent for alterations of TCA cycle enzyme in cancer. Multiple other genes encoding enzymes of the tricarboxylic (TCA) cycle are considered tumor suppressor genes including *SDHB*, *SDHC*, and *SDHD* (Succinate Dehydrogenase subunits B,C,D) [32], [33], [34]. *SDH* subunit mutations have been linked to pheochromocytoma and paraganglioma and more recently to gastrointestinal stromal tumor (GIST) [35]. In addition to mutations, alterations of expression of these genes have been linked to malignancy. Dahia *et al.* demonstrated reduced expression of succinate dehydrogenase subunit B (SDHB) in a subset of pheochromocytomas [36]. More recently, reduced expression of SDHB was identified in large proportion of GISTs without mutations in *SDHB* or other genes commonly mutated in GISTs including *KIT* and *PDGFRA* [35]. Based on these data, a potential unifying theme may be that defects in oxidative phosphorylation, either through mutation or expression changes, have a role in oncogenesis. Recently, Chen *et al.* proposed that oxygen consumption via mitochondrial metabolism may regulate tumor growth by limiting the availability of oxygen for non-mitochondrial activities that are contributory to tumor growth [37].

Of significant interest is our finding that FH overexpression results in reduced migration and invasion of RCC cells. Our data are in concordance with a recent report by Costa *et al.* that demonstrated that *fh* knockdown in immortalized mouse embryonic fibroblasts (iMEFS) resulted in increased motility as compared with untransduced iMEFS [38]. Moreover, the increase in motility was HIF-1α dependent. The role of HIF-2α in their studies could not be determined as they were unable to detect HIF-2α expression in fh deficient iMEFS. Our studies focused on HIF-2α given prior studies that implicate its role in renal carcinogenesis. Given that HIF-2α knockdown in RCC cells inhibits migration, our data indicate that the effects of FH overexpression on migration and invasion are, in part, mediated by effects on HIF-2α. HIF-2α has previously been implicated in the invasive behavior of RCC cells. Moreover the invasive promoting properties of HIF-2α have been studied in 786-O cells, the same cells utilized in this study. Hughes *et al.* demonstrated that HIF-2α knockdown in 786-O cells reduced the expression of multiple integrins that may mediate cell motility and invasion [39]. Petrella *et al.* examined the role of VHL loss in cell invasion [40]. They found that reintroduction of wild type *VHL* into VHL-deficient 786-O cells reduced HIF-2α levels and cell invasion. Conversely, re-expression of HIF-2α in *VHL* reconstituted 786-O cells restored invasive potential. These data indicate a role for HIF-2α in RCC migration and invasion. Additionally, HIF-2α overexpression was found to enhance the growth of RCC xenografts whereas overexpression of HIF-1α was found to inhibit xenograft growth [41]. Correspondingly, separate studies indicate that HIF-2α, but not HIF-1α, contributes to the growth of *VHL* null tumor xenografts [16], [42]. Hence, our data add to the growing body of evidence demonstrating the tumor-promoting effects of HIF-2α expression in RCC.

Despite the recent approval of multiple agents for advanced renal cancer, most patients with advanced renal cancer will eventually succumb to their disease. Hence, the identification of novel signaling pathways will be critical to the development of effective therapeutics. There is now mounting evidence that renal cancer is among the tumors that are representative of the emerging paradigm in cancer biology of metabolic links to malignancy. Our findings with FH suggest a metabolic reprogramming in clear cell renal cancer that promotes expression of tumorigenic factors including HIF-2α via multiple mechanisms. Unraveling the mechanisms by which tumor metabolism is altered and the downstream cellular consequences should provide deep insight in renal cancer biology as well as novel therapeutic strategies.

## Materials and Methods

### Cells

A498, 786-O, and ACHN cells were obtained from the American Type Culture Collection and maintained in DMEM supplemented with 10% heat-inactivated fetal bovine serum at 37°C in a humidified 5% CO_2_ atmosphere. RCC4 cells were kindly provided by P. Ratcliffe (Oxford).

### Chemicals

LY294002 was purchased from Sigma.

### Constructs

The FH-FLAG construct has been previously described [27].

### Immunoblotting

All immunoblot analyses were performed as previously described [27] on whole-cell lysates prepared with the use of radioimmunoprecipitation assay buffer (50 mM Tris-HCl, 150 mM NaCl, 1% Triton X-100, 1% sodium deoxycholate, and 0.1% sodium dodecyl sulfate) supplemented with protease inhibitor cocktail (Roche). Antibodies were obtained from the following commercial sources: GeneTex (FH-immunoblotting), Santa Cruz Biotechnology (FH-immunohistochemistry), Novus (GAPDH, HIF-2α), Sigma (Tubulin, β-Actin), Cell Signaling (total AKT and ser473 phospho AKT).

### Tissue sample quantitative real-time PCR

Biospecimens for RNA analysis were obtained from the Cooperative Human Tissue Network of the NCI/NIH. Total RNA was reverse transcribed using the High-Capacity cDNA Archive kit (Applied Biosystems). cDNA was then used as template with Applied Biosystems' assays-on-demand 20× assay mix of primers and Taqman probes. Forty amplification cycles were done on the Applied Biosystems Prism 7900 sequence detector. Fold change values between tumor and normal samples were calculated using the Δ*C*_t_ method with normalization to 18S rRNA levels. As a statistical test we used the Mann-Whitney paired non-parametric test to compare FH expression in tumor versus normal adjacent tissue (implemented in GeneSpring, Agilent).

### Immunoblotting and immunohistochemistry of human tumor specimens

Tumor samples and normal corresponding tissue from patients with RCC were obtained from the Department of Urology at the University of Texas Health Science Center at San Antonio. The tumors for this study were histologically classified as clear cell renal carcinoma by a genitourinary pathologist. The collection and handling of human samples was performed according to a protocol approved by the Institutional Review Board of the University of Texas Health Science Center at San Antonio. Based on the protocol, samples were obtained in a deidentified fashion from patients undergoing surgical resection for renal cancer. As samples were obtained and analyzed in an anonymous fashion, patient consent was not required.

### Wound healing assay

Cells were allowed to grow to near confluence in 60 mm dishes. A uniform scratch was then made down the center of the plate using a 200 microliter micropipette tip, followed by washing twice with PBS. The same marked field of the scratch wound was photographed using an Olympus light microscope (4× objective) at the indicated time points. The width of the scratch wound was measured at three different areas with Q-Capture pro software. Quantified data represent the mean +/− S.D. from at least two independent experiments.

### Migration assay

786-O subclones cells (1×10^4^) were seeded on an 8 µM pore size Thin-cert for 24 well plates (Greiner Bio-One) in serum free media. Seven hundred fifty microliters of 10% FBS medium was added to the bottom chamber as chemotractant. After 48 hrs, cells on the top of the membrane were removed with a cotton swab. The migrated cells at the bottom side were washed with PBS, fixed with 70% ethanol and stained using 0.1% Crystal violet to visualize the migrated cells. Migrated cells attached to the lower side of the membrane were enumerated using a light microscope at 10× magnification. Counts represent the average cell number of ten microscopic fields.

### Invasion assay

Cell invasion was determined by invasion assay (membrane coated with a layer of Matrigel extracellular matrix proteins) according to the manufacturer's instructions. Cells were seeded in serum-free medium into the upper chamber and invaded toward the bottom chamber containing a 10% FBS medium as the chemotractant. Membranes were processed in a similar fashion as the migration assay.

### RNA interference

For FH and HIF-2α knockdown, cells were transfected with pooled siRNA reagent (Thermo Fisher) with the Amaxa Nucleofector system according to the manufacturer's protocol. Cells were harvested at 48–72 hours following transfection. A non-targeting scramble siRNA pool was used as a negative control (Thermo Fisher).
